# Embryonic cells contribute directly to the quiescent stem cell population in the adult mouse mammary gland

**DOI:** 10.1186/s13058-014-0487-6

**Published:** 2014-12-03

**Authors:** Kata Boras-Granic, Pamela Dann, John J Wysolmerski

**Affiliations:** Section of Endocrinology and Metabolism Department of Internal Medicine, Yale University School of Medicine TAC S131, New Haven, 06520-8020 CT USA

## Abstract

**Introduction:**

Studies have identified multi-potent stem cells in the adult mammary gland. More recent studies have suggested that the embryonic mammary gland may also contain stem/progenitor cells that contribute to initial ductal development. We were interested in determining whether embryonic cells might also directly contribute to long-lived stem cells that support homeostasis and development in the adult mammary gland.

**Methods:**

We used DNA-label retention to detect long label-retaining cells in the mammary gland. Mouse embryos were labeled with 5-ethynl-2′-deoxyuridine (EdU) between embryonic day 14.5 and embryonic day 18.5 and were subsequently sacrificed and examined for EdU retention at various intervals after birth. EdU retaining cells were co-stained for various lineage markers and identified after fluorescence activated cell sorting analysis of specific epithelial subsets. EdU-labeled mice were subjected to subsequent 5-bromo-2′-deoxyuridine administration to determine whether EdU-labeled cells could re-enter the cell cycle. Finally, EdU-labeled cells were grown under non-adherent conditions to assess their ability to form mammospheres.

**Results:**

We demonstrate embryonically-derived, long label-retaining cells (eLLRCs) in the adult mammary gland. eLLRCs stain for basal markers and are enriched within the mammary stem cell population identified by cell sorting. eLLRCs are restricted to the primary ducts near the nipple region. Interestingly, long label retaining cells (labeled during puberty) are found just in front of the eLLRCs, near where the ends of the ducts had been at the time of DNA labeling in early puberty. A subset of eLLRCs becomes mitotically active during periods of mammary growth and in response to ovarian hormones. Finally, we show that eLLRCs are contained within primary and secondary mammospheres.

**Conclusions:**

Our findings suggest that a subset of proliferating embryonic cells subsequently becomes quiescent and contributes to the pool of long-lived mammary stem cells in the adult. eLLRCs can re-enter the cell cycle, produce both mammary lineages and self-renew. Thus, our studies have identified a putative stem/progenitor cell population of embryonic origin. Further study of these cells will contribute to an understanding of how quiescent stem cells are generated during development and how fetal exposures may alter future breast cancer risk in adults.

**Electronic supplementary material:**

The online version of this article (doi:10.1186/s13058-014-0487-6) contains supplementary material, which is available to authorized users.

## Introduction

**Figure 1:**
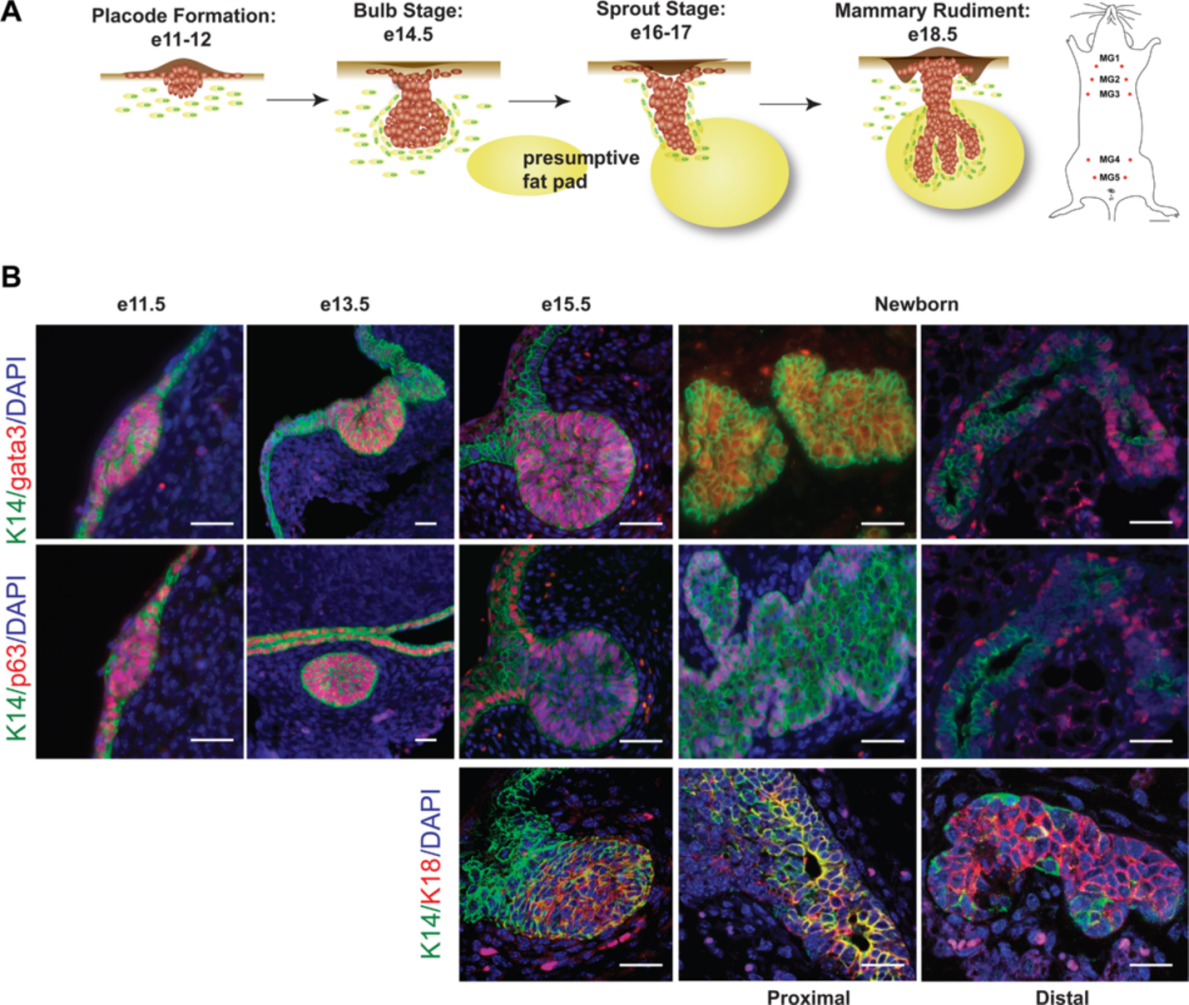
**Luminal and myoepithelial lineage marker expression during embryonic mammary gland development. (A)** Schematic representation of embryonic mammary rudiment formation in the female from e11.5 to birth. Five pairs of mammary glands form in the female mouse. **(B)** Immunostaining for K14 (green) and Gata3 (red) (top), K14 (green) and p63 (red) (middle), and K14 (green) and K8 (red) (bottom) in WT MG at e11, e13, e15, and newborn (one day old). Scale bars, 40 μm. e, embryonic day; MG, mammary gland; WT, wild type.

In mice, mammary gland development begins around embryonic day 10.5 (e10.5) with the formation of bilateral mammary lines between the fore and hind limb buds along the ventral-lateral borders of the embryo. Cells within the mammary line coalesce into five distinct pairs of placodes (three thoracic and two inguinal). Over the next several days, each mammary placode expands and invaginates into the underlying mesenchyme to form a mammary bud (Figure 1A). Mammary rudiments have very low proliferative activity between e11.25 and e13.5 and the initial phases of mammary development are thought to rely on cell migration from the epidermis rather than proliferation of mammary epithelial cells [1]-[3]. Active proliferation within the mammary epithelium begins at e14.5 [4]. By e15.5, the distal end of the mammary bud begins to elongate into the underlying dermal mesenchyme to form a sprout. The sprout grows downward into the mammary fat pad, an adipocyte-rich stromal compartment and begins to branch, forming the rudimentary ductal tree by e18.5. By birth, the mammary epithelium consists of a primary duct and about 10 to 15 branches located within the proximal end of the nascent mammary fat pad.

The postnatal mammary gland continues to undergo periodic development and remodeling. The nascent ductal system grows isometrically until puberty when ductal morphogenesis accelerates in response to hormonal cues. At puberty, terminal end buds (TEBs) form at the tips of mammary ducts [5]. TEBs are the predominant sites of epithelial proliferation during puberty, as the ducts invade the surrounding stromal tissue and elongate to the distal end of the fat pad. In virgin animals, cyclical development and regression of small ductules and alveolar buds occurs along the epithelial ducts with each estrous cycle. During pregnancy, these buds further develop into fully formed alveoli that fill the fat pad in preparation for milk production during lactation [5]-[7]. This process involves vigorous proliferation and the secretory differentiation of a large number of new epithelial cells. Once lactation ceases, the alveoli regress during involution, most of the newly generated epithelial cells die and the mammary gland returns to a resting state. The cycle of pregnancy, lactation, and involution can repeat itself multiple times during the reproductive lifespan of an animal, suggesting the presence of stem/progenitor cells to supply each new cycle of expansion.

By definition, tissue stem cells have the capacity to generate all cell types of the tissue in which they reside and are able to self-renew in order to support long-term homeostasis of an organ. The existence of a population of stem cells in the adult mammary gland was initially shown by the ability of any fragment of the mammary gland to reconstitute an entire gland upon transplantation as well as the reproduction of genetic chimerism upon transplantation of fragments of a chimeric gland [8],[9]. More recently, the existence of individual stem cells was demonstrated by the ability of a single cell to regenerate the entire mammary epithelium upon transplantation [10],[11]. These experiments implicate the existence of a multi-potent cell that can give rise to myoepithelial, luminal and alveolar lineages. However, recent lineage tracing studies disagree on whether multi-potent cells actually give rise to the various mammary epithelial lineages during development and reproductive cycles *in vivo,* or whether lineage-restricted progenitor cells are induced to behave in a multi-potent manner in transplantation studies [12]-[14].

One potential source of multi-potent mammary stem cells may be the embryonic mammary bud [15]. Intact mammary buds from as early as e12 can give rise to an entire mammary gland when transplanted into the cleared fat pad of pubertal mice. In contrast, when dissociated into single cells and grafted into cleared fat pads, the repopulating potential of the embryonic mammary epithelium was found to be very low before e15.5. In these experiments, significant stem cell or repopulating activity was only detected after e16.5 and increased dramatically by e18.5 [16],[17]. Lineage tracing studies also support the existence of bipotent mammary stem cells during late embryogenesis (e17.5) [12]. The relationship between embryonic and adult mammary stem cells has yet to be fully determined. None of these prior studies have carefully examined whether embryonic mammary stem cells are maintained during postnatal development and/or whether they contribute to the pool of multi-potent and/or unipotent stem cells within the adult mammary epithelium.

The fact that stem cells may remain quiescent for long periods has been exploited to identify putative stem/progenitor cells in several tissues. Label retention relies on progressive dilution of a DNA label in actively dividing cells. By contrast, stem cells, which divide infrequently, retain label for long periods of time, much longer than the surrounding non-stem cells. The administration of DNA labels during puberty has identified long label-retaining cells (LLRC) in the epithelial ducts in adult virgin mice and in alveolar structures during pregnancy [18]-[22]. Slowly cycling cells have also been identified using either PKH26 labeling of mammospheres *in vitro* [23] or through an inducible histone 2b promoter linked to a GFP reporter (K5tTA-H2b-GFP) [24]. In the current study, we employed long-term label retention as a method to identify quiescent stem cells that came from the embryonic mammary gland. Our data support the presence of long-lived, multi-potent cells that originate during embryonic mammary development and that contribute to the homeostasis of the adult mammary gland.

## Methods

### Animals

CD1 mice, purchased from Charles River (Wilmington, MA, USA), were bred and maintained in our animal facility according to institutional guidelines. All experiments were approved by Yale University’s Institutional Animal Care and Use Committee.

### Nuclear DNA labeling

For label retention studies, pregnant mice were administered an intraperitoneal (IP) injection of 5-ethynyl-2-deoxyuridine (EdU, 50 mg/kg of mouse) twice daily from e14.5 to e18.5. For cell proliferation analysis, 5-bromo-2′-deoxyuridine (BrdU, 30 mg/kg of mouse) was injected IP twice daily for five days at the specified stages after birth (day 1 to 5), three weeks (21 days to 26 days), eight weeks (56 days to 61 days), twelve weeks (84 days to 89 days) and early-mid pregnancy (day 5 to day 12).

### Immunofluorescence analysis

Whole embryos and mammary glands were fixed in 4% paraformaldehyde (PFA) at 4°C for 12 hours. Ventral skins containing mammary buds were dissected and embedded. Mammary buds were identified by serial sectioning, as described previously [25]. Immunohistochemistry was performed using standard techniques [26]. Antigen retrieval was accomplished by heating sections in 10 mM citrate, under pressure. Sections were incubated overnight at 4°C with antibodies directed against estrogen receptor (ER), progesterone receptor (PR), K14, K18, Gata3 and p63. Staining was detected using Alexa Fluor 488-conjugated goat anti-mouse, Alexa Fluor 555-conjugated goat anti-rabbit and streptavidin-conjugated Alexa Fluor 647-secondary antibodies (Invitrogen, Grand Island, NY,USA) for immunofluorescence. EdU incorporation was assessed using the Click-IT EdU system (Invitrogen) according to the manufacturer’s instructions. Fluorescence was detected using a Cy3filter. EdU-positive and total cells were evaluated in a total of three to five #4 mammary glands harvested from at least three different mice. BrdU incorporation was assessed using anti-BrdU antibodies. The percentage of embryonically derived long label retaining cells (eLLRCs) or proliferating cells was calculated and statistical significance was determined by performing Student’s *t*-test. The significance of the results was determined by performing a one-tailed Student’s *t*-test using Prism v4.0b software (GraphPad Software).

### Mammary cell isolation and flow cytometry

We dissected the #4, inguinal mammary glands from 10 to 20 female mice who had been exposed to EdU *in utero* (the number of mice varied based on the number of female offspring after embryonic labeling) and placed them in a 50-ml tube containing dissociation medium (one part 10X gentle collagenase/hyaluronidase and nine parts Epicult-B medium supplemented with 5% fetal bovine serum (FBS) and gentamicin) (StemCell Technologies, Vancouver, Canada). Typically for mammosphere experiments, the portion of the #4 gland from the nipple region to the lymph node was used for cell isolation to enrich for EdU+ cells since immunofluorescence examination did not identify eLLRCs beyond the lymph node. The tissue was digested for six hours at 37°C in a 5% CO_2_ incubator. The resultant organoid pellet was resuspended first in NH_4_Cl for five minutes and spun down, followed by treatment with 0.25% trypsin-ethylenediaminetetraacetic acid (EDTA) for one to three minutes and spun down, and then treated with 5 mg/mL dispase and 0.1 mg/mL DNase I for one to three minutes and spun down. The cell suspension was then filtered through a 40-micron mesh and labeled with various antibodies for further purification. All incubations and washes were performed in Hank’s balanced salt solution (HBSS) supplemented with 2% FBS. Cells were first incubated with anti-CD16/CD32 (Fcγ III/II receptor) for 10 minutes on ice to reduce Fc receptor-mediated binding, followed by a 15 minute incubation on ice with the biotinylated CD31/CD45/Ter119 antibody cocktail. After washing, cells were incubated with anti-CD24-PE, anti-CD49f-FITC, and/or streptavidin-APC on ice for 10 minutes. After washing, cells were sorted using fluorescence activated cell sorting (FACS) (MoFlo, BD Bioscience, San Jose, CA, USA) for these cell surface markers. We verified the purity of sorted populations by re-sorting an aliquot of the isolated cells for the same surface markers used for their isolation and found that >95% of the cells had the expected cell-surface phenotype. Cells isolated by FACS were used for (1) mammosphere assays as described below or (2) Click-It assay on cytospins of freshly sorted cells or (3) FACS analysis for EdU incorporation. For FACS analysis of EdU, single sorted cells were fixed with 4% PFA, stained using the Click-It assay for Alexa-647 and re-analyzed by FACS (MoFlo, BD Bioscience).

### Mammosphere assay

For mammosphere formation, we used the FACS sorted population of CD49f^high^CD24^+^ MaSC-enriched basal cells as previously described [11]. Cell suspensions were plated in six-well ultralow attachment tissue culture plates (Corning, NY, USA) with mouse EpiCult-B complete medium supplemented with 2% B27 (Gibco, Grand Island, NY, USA), 20 ng/mL basic fibroblast growth factor (bFGF), 20 ng/mL epidermal growth factor (EGF), 10 μg/mL heparin, 10 μg/mL insulin, 1 μg/mL hydrocortisone and 50 μg/mL gentamicin (referred to as mammosphere medium) [27]. The medium was made semi-solid by the addition of 1% methylcellulose (R&D Systems, Minneapolis, MN, USA) to prevent cell aggregation and to ensure that mammospheres were derived from single cells as previously documented [28]. Sorted basal cells were plated at a density of 200 cells/cm^2^. Cells were cultured at 37°C in 5% CO_2_. After 10 days in culture, mammospheres were collected by gentle centrifugation (200 × g) and agitated with a P200 micropipette to segregate individual spheres to split for embedding or secondary cultures. For embedding, mammospheres were fixed with 4% PFA and embedded in paraffin for subsequent immunostaining. For secondary cultures, mammospheres were dissociated enzymatically (five minutes in 1:1 trypsin/(Dulbecco’s) modified Eagle’s medium ((D)MEM) solution at 37°C) and mechanically by passing through a 25G needle. Single cell suspensions were re-plated at a density of 200 cells/cm^2^ in 1% methylcellulose for subsequent passages. Cells were cultured at 37°C in 5% CO_2_. After seven days in culture, secondary mammospheres were collected by gentle centrifugation (200 × g), fixed with 4% PFA and embedded in paraffin blocks for further staining.

### Image acquisition and processing

High-magnification images were acquired using an inverted LSM710 laser scanning confocal microscope (Zeiss) through a 63× oil objective or Axiovision Zeiss through 20× and 40× objectives. RGB images were assembled using ImageJ software and z stacks were processed for three-dimensional visualization.

## Results

### Lineage marker expression during embryonic mammary gland

Previous studies have suggested that cells in the embryonic mammary gland express both luminal and basal markers [29]-[32]. In order to confirm that embryonic mammary cells share aspects of both lineages, we examined the expression of the basal markers, K14 and p63, and the luminal marker, Gata3, from the onset of placode development (e11) to the formation of the rudimentary ductal tree (e18). We found that basal and luminal markers were co-expressed in the mammary placodes at e11 and the buds at e13 (Figure 1B). At e15, K14, p63, Gata3 and another luminal marker, K18, were co-expressed in the main part of the bud, but K18 and Gata3 were not expressed within the neck region of the bud. While the bilayered ductal system is not fully established until after birth, basal and luminal markers began to segregate during late embryogenesis. Specifically, expression of the basal marker, p63, becomes localized around e15.5 to the outer cell layer of the developing ducts, the site of future myoepithelial cells (Figure 1B). In contrast, K14 expression was heterogeneous during late embryogenesis and, at birth, was significantly reduced at the ends of the rudimentary ducts compared to the nipple region. Interestingly, Gata3 was expressed in the basal layer of cells located in the distal rudimentary ductal tree and in both basal and luminal locations in the nipple region at birth. Furthermore, in newborn pups, K14 and K18 were coexpressed in cells of the primary duct close to the nipple region, but they became more clearly segregated from one another at the ends of the ducts (Figure 1B). These data suggest that early embryonic mammary cells are bipotent and that the segregation of p63 to the basal cell layer may be one of the first distinctions between luminal and basal cell lineages. Furthermore, the segregation of basal and luminal marker expression at birth is most evident at the distal ends of the epithelial ducts and appears to occur coincident with their outgrowth from the mammary bud.

### DNA labeling during embryogenesis identifies eLLRCs in the adult mammary gland

**Figure 2:**
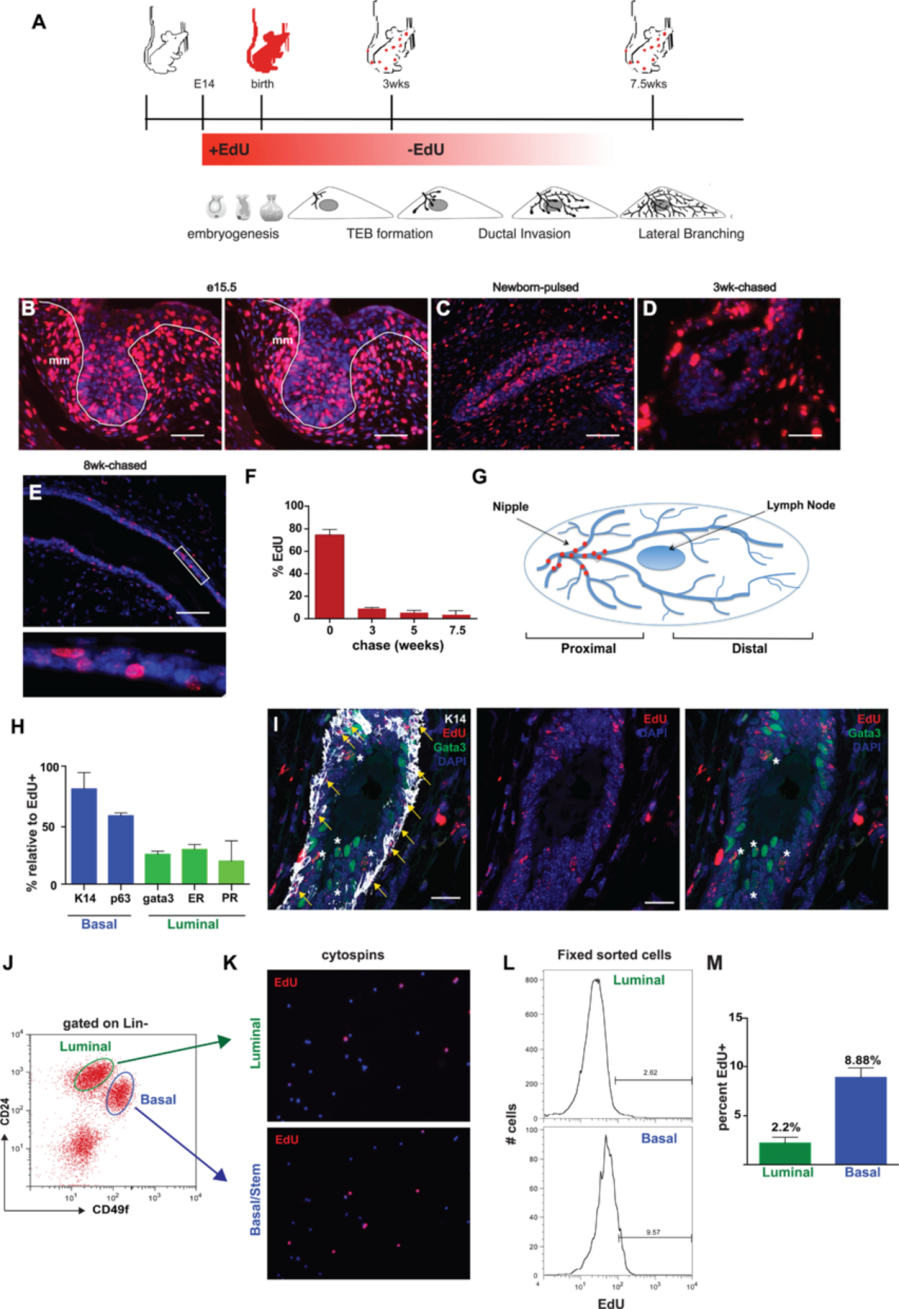
**Embryonically EdU-labeled cells persist in the mammary ducts of adult mice. (A)** Schematic protocol to study embryonic label retention in the mouse mammary gland. **(B)** EdU label incorporation was examined in paraffin sections from e15 embryos labeled for 24 hours. EdU+ cells are located in the epithelial bud and surrounding mesenchyme. Paraffin sections stained for EdU from MGs of newborn **(C)**, three-week-old **(D)**, and eight-week-old **(E)** mice after EdU administration between e14.5 and e18. L = lumen. **(F)** Percentage of EdU-labeled cells at various time-points after embryonic administration. (n = 3 to 5 mice/stage) **(G)** Diagram of LLRC location in eight week MG. **(H)** Percentage of EdU+ cells that express basal or luminal lineage markers in eight week MGs. (n = 1,000 cells). Bars represent SE. [See Additional file 2]. **(I)** Paraffin section of proximal portion of 10-week chased MG co-stained for EdU, K14 and Gata3. Edu + cells (red) are located predominantly in the basal layer of the ducts and are a subpopulation of the K14-positive cells (white; see yellow arrows). EdU+ cells are also located in the luminal layer of the ducts and are a rare subpopulation of the Gata3-positive cells (green; see white asterisks). (J-M) Summary of flow cytometry data. **(J)** MGs were isolated from eight-week-old EdU pulse-chased mice and analyzed by flow cytometry for expression of cell surface markers, CD24 and CD49f. EdU+ cells represent 1.2% of total mammary epithelial cells Lin-CD24+ [See Additional file 1]. **(K)** Immunofluorescence analysis for EdU+ cells in cytospins of sorted Lin^-^CD24^+^CD49f^low^ luminal and Lin^-^CD24^+^CD49f^high^ basal cells. **(L)** Representative histogram FACS plot of sorted and fixed populations of Lin^-^CD24^+^CD49f^low^ luminal and Lin^-^CD24^+^CD49f^high^ basal cells. **(M)** Average percentage of EdU+ cells in Lin^-^CD24^+^CD49f^lo^ luminal (2.2%) and Lin^-^CD24^+^CD49f^high^ basal (8.88%) cell populations. Data represent mean ± SEM of three independent experiments. Scale bars, 20 μm, mm = mammary mesenchyme. e, embryonic day; EdU, 5-ethynl-2′-deoxyuridine; FACS, fluorescence activated cell sorting; LLRC, long label retaining cells; MG, mammary gland; SE, standard error; SEM, standard error of the mean.

Given that cells in the embryonic mammary buds co-express markers of both epithelial lineages (Figure 1) and in light of the ability of the mammary bud to generate a functional mammary gland when transplanted, we postulated that these structures contain mammary stem/progenitor cells [33]. Furthermore, we also reasoned that these stem/progenitor cells not only proliferate to support the outgrowth of the embryonic gland but that some proportion of these cells become quiescent to contribute to subsequent phases of development during the life cycle of the mammary gland. In order to identify quiescent or slowly cycling embryonic-derived stem/progenitor cells, we used pulse–chase labeling with the thymidine analog, EdU. Pregnant mice were pulsed twice daily with EdU from day 14.5 through day 18 in order to label dividing cells within the embryonic mammary anlage at the initiation of ductal outgrowth (Figure 2A). Mice were subsequently allowed to deliver and the mammary glands of female offspring were analyzed by immunostaining and flow cytometry to identify EdU-labeled cells.

We first evaluated embryos at e15.5 after one day of EdU labeling (pulsed e14.5 to e15.5) and at birth, after five days of EdU labeling (pulsed e14.5 to e18). As shown in Figure 2B and C, most, but not all, of the epithelial cells were EdU-positive after the initial labeling period. Specifically, at the end of the embryonic pulse period (e14.5 to e18), approximately 75% of mammary epithelial cells were labeled with EdU (Figure 2F). We next examined the frequency of cells that retained EdU over a period of eight weeks, during which time the ducts grew to fill out the mammary fat pad. As cells actively proliferate during ductal growth, they will progressively dilute the DNA label and the number of labeled cells will rapidly decline. By contrast, stem or progenitor cells that cycle only occasionally or that divide asymmetrically and retain their template DNA strand will continue to be EdU-positive [21]. We found that the abundance of eLLRCs decreased during ductal development. At the onset of puberty at three weeks of age, 8.6% of epithelial cells remained labeled but by eight weeks, only 1.1% of epithelial cells were EdU-positive (Figure 2F). eLLRCs were most common in the proximal portion of the gland, near the origin of the ducts at both time points, although EdU-positive cells were evident in the TEBs at three weeks of age (Figure 2D). However, at eight weeks of age, eLLRCs were only seen in the proximal third of the mammary gland, principally in the primary ducts clustered toward the nipple area (Figure 2E,G); no eLLRCs were detected in the remaining TEBs or in cells at the tips of the ductal tree.

We next characterized the phenotype of eLLRCs in the epithelial ducts. At eight weeks, eLLRCs were noted in both basal and luminal locations in the epithelium (Figure 2H and Additional file 1: Figure S1). Staining for basal or luminal lineage markers and EdU revealed that the majority of the eLLRCs were in the basal lineage. As seen in Figures 2H and S1, at 7.5 weeks, 88% of the EdU-positive cells were also K14-positive and 60% were p63-positive. In addition, 26% of eLLRCs were positive for the luminal marker, Gata3, while 34% of the EdU-positive cells stained for the ER and 28% stained for the PR. These data suggest that more than 10% of eLLRCs might be positive for both luminal and basal lineage markers. However, these percentages were derived by staining different tissue sections for either basal or luminal markers and we did not detect any specific eLLRCs that co-stained for both lineages in sections when we simultaneously stained for both basal and luminal markers (Figure 2I). As shown in Figure 2I, in the primary duct near the nipple region, EdU-positive cells stained for either K14 or Gata3, but not both.

Based on the hypothesis that eLLRCs comprise a population of potential mammary stem/progenitor cells (MaSC), we investigated the correlation between EdU label retention and the expression of previously defined MaSC cell surface markers, CD24 and CD49f [11]. Using FACS analysis, we detected a small population (1.2%) of EdU+ eLLRCs present in total mammary epithelial cells (MECs) sorted from eight-week-old, embryonically labeled mammary glands [see Additional file 2: Figure S2E]. Notably, we also confirmed the presence of eLLRCs from single day pulsed e15, e16 and e17 mammary glands [see Additional file 2: Figure S2]. Based on our immunofluorescence analysis showing that eLLRCs were located proximal to the lymph node in the #4 mammary gland, we restricted further analysis to the proximal region of the mammary gland (nipple to the lymph node). Murine MECs were sorted into either CD49f^high^CD24^+^ MaSC-enriched basal cells, or the CD49f^low^CD24^+^ luminal progenitors and differentiated luminal cells, and each of these cell populations was subsequently further analyzed for EdU label retention by immunofluorescence (Figure 2 J,K) or FACS for EdU-positive cells in five independent experiments (Figure 2J,M). On average, EdU-positive cells represented 2.2 ± 0.41% of the sorted luminal cells and 8.8 ± 0.685% of the sorted basal cells. Percentages of EdU-positive cells across the individual experiments varied to some degree; however, in all experiments, the stem cell-enriched population, Lin^−^CD49f^high^CD24^+^, contained the highest percentage of EdU-positive cells, consistent with the enrichment of quiescent or slowly-cycling eLLRCs within the MaSC-enriched fraction of the adult mammary epithelial cells [7],[22],[24].

### eLLRCs are deposited near the edge of the growth front at the time of labeling

**Figure 3:**
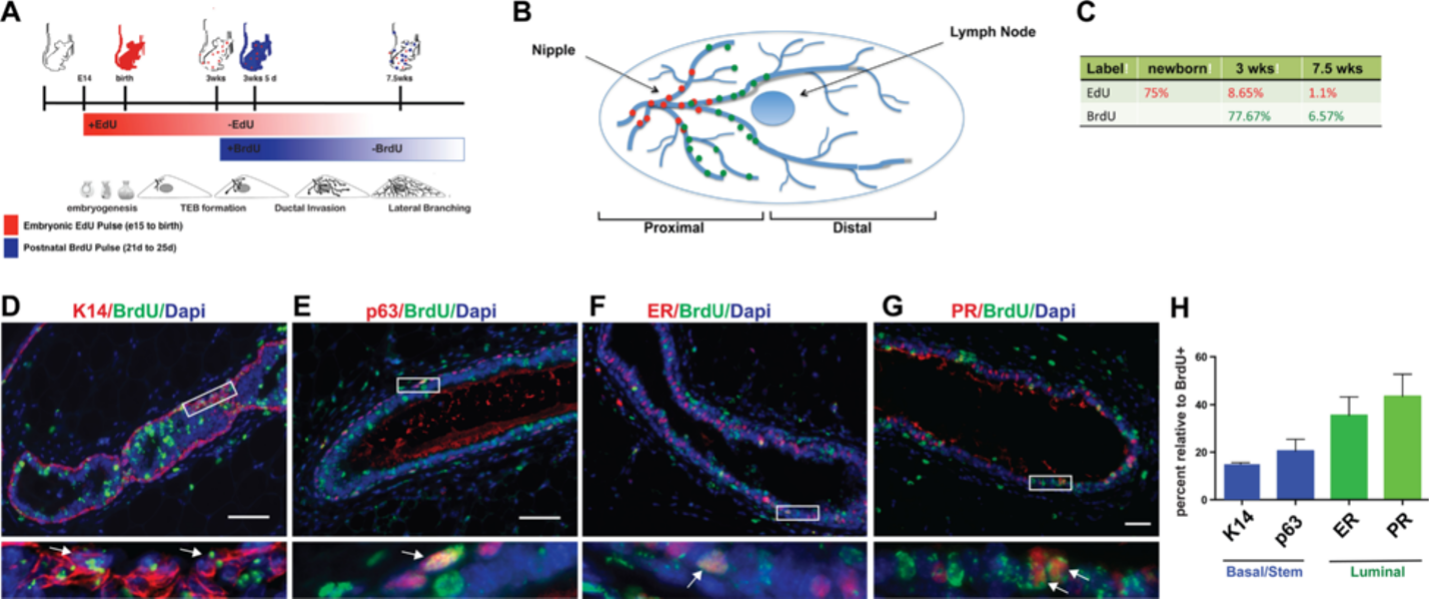
**Embryonic EdU labeling followed by pubertal BrdU labeling demonstrates overlapping but distinct LLRC populations. (A)** Scheme for sequential embryonic labeling with EdU from e14.5 to e18.5 followed by pubertal labeling with BrdU from day 21 to 25. **(B)** Diagrammatic representation of the relative localization of EdU-retaining and BrdU-retaining epithelial cells in the adult mammary gland at eight-weeks of age. EdU-retaining cells localized to the main ducts in the nipple region, while BrdU cells are localized between the EdU-positive cells and the lymph node. **(C)** Percentage of EdU-positive and BrdU-positive cells at different time points following labeling. Representative sections co-stained for BrdU and the myoepithelial markers K14 **(D)** or p63 **(E)**, or the luminal markers, estrogen receptor **(F)** or progesterone receptor **(G)**. Boxes represent areas of inset. Arrows in insets point to double-labeled cells. **(H)** Percentage of BrdU-positive cells that also stain for K14, p63, ER or PR. BrdU, 5-bromo-2′-deoxyuridine; e, embryonic day; EdU, 5-ethynl-2′-deoxyuridine; ER, estrogen receptor; LLRC, long label retaining cells.

Given that embryonically EdU-labeled cells were restricted to the proximal portion of the mammary gland near the nipple region, it suggested to us that quiescent eLLRCs might be deposited in the ducts behind the active growth front at the time of labeling. In order to test this idea, we performed sequential labeling first with EdU from e14.5 to e18 and then with BrDU for five days beginning at the initiation of puberty at three weeks of age (21 days) (Figure 3A). As depicted in Figure 3B, at the end of the BrDU pulse period (25 days), approximately 77% of epithelial cells were BrdU-positive based on immunofluorescence analysis. We then examined the mammary glands for EdU and BrdU labeling at eight weeks of age, five weeks after BrdU labeling. The percentage of BrdU-positive cells decreased to 6.5%. In contrast to the EdU label-retaining cells, the BrdU label-retaining cells were enriched in the luminal rather than the basal compartment. Twenty percent of BrdU-positive cells expressed p63 and 14% expressed K14 while one third were positive for the luminal hormone receptors, PR and ER (Figure 3E). These results are in agreement with previously published data identifying adult LLRCs [18],[19],[21]. Strikingly, BrdU label-retaining cells were enriched in the proximal portion of the mammary gland just before the lymph node (Figure 3B). While there was some overlap with the EdU-positive cells, the BrdU-positive cells were mostly between the EdU-positive cells and the lymph node, approximately at the location where the ends of the ducts had been at the time that the BrDU pulse was given. These data demonstrate that a subset of cells near the active growth front can become LLRCs suggesting that quiescent stem/progenitor cells are deposited along the elongating ducts as a function of active morphogenesis occurring at the leading edge.

### A subset of embryonically derived LLRCs are mitotically active during postnatal development

**Figure 4:**
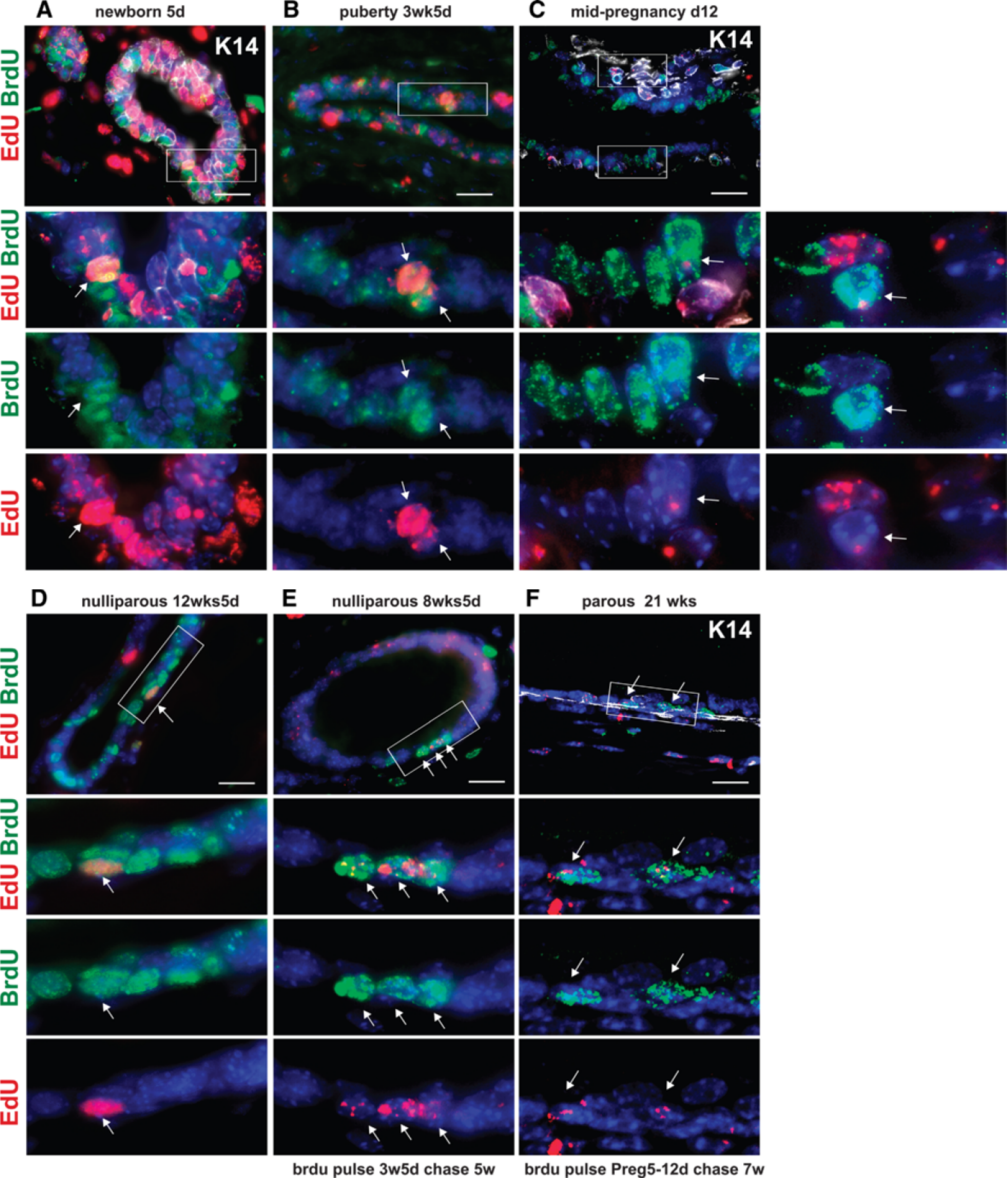
**A subset of eLLRCs re-enter the cell cycle during postnatal development and tissue remodeling.** Embryonically EdU-labeled (e14.5 to e18) animals were administered BrdU for five days at different stages of postnatal development. Paraffin sections of mammary glands at **(A)** five days (newborn), **(B)** three weeks and five days (puberty), **(C)** mid-pregnancy (12.5 days) and **(D)** four months (virgin-nulliparous) were co-stained for EdU and BrdU. K14 staining is included in some samples to aid in tissue structure visualization. Double-labeled EdU + BrdU+ cells (see white arrows) were evident in glands analyzed at all time points. In addition, a cohort of female mice that received a second BrdU pulse at puberty or pregnancy was also chased for an additional five to seven weeks **(E,F)**. Examination of BrdU pulse-chased mammary glands demonstrated double-labeled LRCs at the end of puberty (eight weeks of age) **(E)**, and occasional rare EdU + BrdU + LRCs at the end of involution **(F)**. White arrows denote double-labeled cells. Scale bar, 20 μm. Rectangles highlight areas shown at higher magnification. BrdU, 5-bromo-2′-deoxyuridine; e, embryonic day; EdU, 5-ethynl-2′-deoxyuridine; eLLRCs, embryonically derived long label retaining cells; LRCs, label retaining cells.

eLLRCs could represent quiescent, slowly cycling stem/progenitor cells or they might simply represent post-mitotic, long-lived, differentiated epithelial cells. We postulated that during phases of active development and proliferation, a quiescent stem/progenitor cell would be activated and re-enter the cell cycle to produce progeny cells and to self-renew. In order to distinguish between quiescence and terminal differentiation, we administered BrdU to the embryonically EdU-labeled mice for a period of five days at different stages of development, including neonatal development, pubertal development and pregnancy (Figure 4). After acute labeling with BrdU, we examined serial sections for EdU-positive cells that also incorporated BrdU, indicating re-entry into the active phase of proliferation. At each of these stages, a small subset of eLLRCs was mitotically active as seen by the presence of double-labeled cells (EdU^+^BrdU^+^) (Figure 4A-C). We also treated adult, nulliparous mice (12 weeks old) with BrdU and found that some EdU-positive cells incorporated BrdU during tissue homeostasis, presumably as a result of cyclical estrous cycle changes (Figure 4D). Finally, we examined whether any of these activated eLLRCs (EdU^+^BrdU^+^) remained within the gland over time (Figure 4E and F). Animals labeled with EdU during embryogenesis and subsequently pulsed with BrdU either at three weeks of age (21 days to 25 days) or during pregnancy (day 5 to day 12) were chased for an additional five and seven weeks, respectively. Mammary glands were examined at eight weeks of age when the ducts filled the fat pad or at the end of involution after regression of the lactating gland (Figure 4E and F). In resting glands chased from pregnancy, there was overall weaker EdU label in epithelial cells given the extensive proliferation, differentiation and regression which occurred during the chase period. We observed a small number of double-labeled EdU^+^BrdU^+^ LLRCs both at eight weeks of age after pubertal BrdU labeling and seven weeks after labeling during mid-pregnancy, demonstrating that proliferating eLLRCs could become quiescent once again, suggesting self-renewal of long-lived stem/progenitor cells.

**Figure 5:**
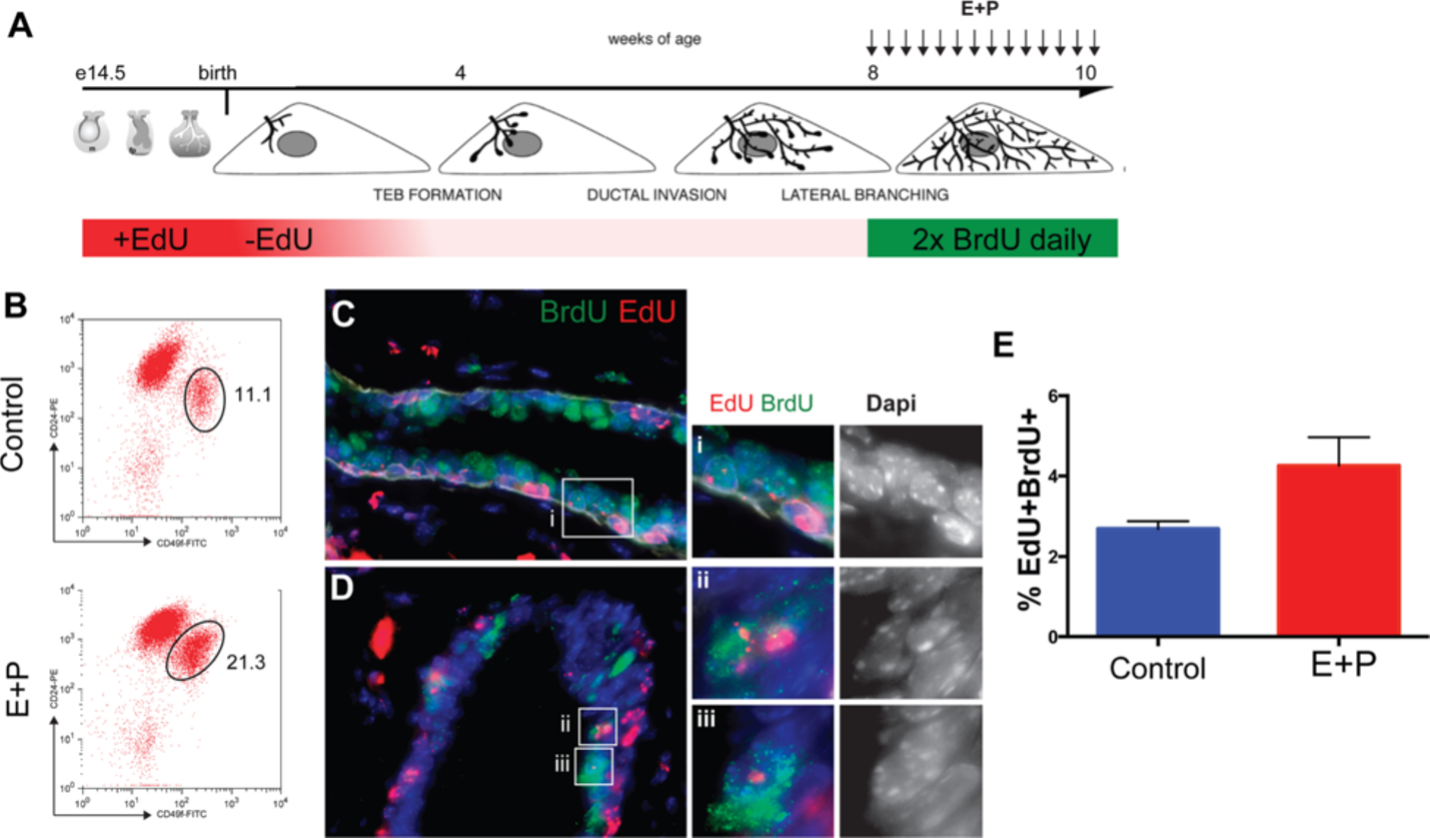
**Hormone-dependent activation of a subset of eLLRCs in the adult mammary gland. (A)** Diagrammatic representation of protocol used to examine proliferation of embryonic LLRCs during hormone treatment in adult virgin mice. Embryonically EdU-labeled adult mice were injected daily for two weeks with estrogen and progesterone (E + P) or vehicle and with BrdU twice/day. **(B)** FACS profiles of luminal CD24^+^CD49f^low^ and basal CD24^+^CD49f^high^ cell populations isolated from either control or E + P treated 10-week old mammary glands. **(C** **,** **D)** Immunofluorescent staining of paraffin sections for EdU and BrdU. Scale bar represent 20 μm. **(E)** Histograms showing quantification of the eLLRCs that proliferate after hormone treatments. Hormone treatment resulted in a 1.7-fold increase in the number of EdU+ LLRCs labeled with BrdU. Data represent mean ± SEM of n = 3 mice each. BrdU, 5-bromo-2′-deoxyuridine; EdU, 5-ethynl-2′-deoxyuridine; eLLRCs, embryonically derived long label retaining cells; SEM, standard error of the mean.

During puberty and pregnancy, mammary development and epithelial cell proliferation are regulated by the ovarian hormones, estrogen and progesterone. Furthermore, it has been shown that MaSCs are hormonally regulated and proliferate in response to estrogen and progesterone [34],[35]. To examine whether estrogen and progesterone were capable of activating eLLRCs, eight-week-old nulliparous mice, previously labeled with EdU during embryogenesis, were injected once daily with estradiol and progesterone (E + P) or vehicle, and twice daily with BrdU for a period of two weeks (Figure 5). FACS analysis confirmed an increase in the number of basal/MaSC cells in mice treated with E + P hormones, consistent with previous reports [35]. Furthermore, we found that hormone treatment increased the number of double-labeled EdU^+^BrdU^+^ LLRCs by 1.7-fold (Figure 5C-D), demonstrating that the eLLRCs divided in response to estrogen plus progesterone. Collectively, these data demonstrate that eLLRCs can re-enter the cell cycle during epithelial development, become quiescent again after dividing and proliferate in response to ovarian hormones. These findings support the idea that eLLRCs are long-lived stem/progenitor cells rather than terminally differentiated ductal cells.

### Embryonically derived LLRCs contribute to primary and secondary mammosphere formation

**Figure 6:**
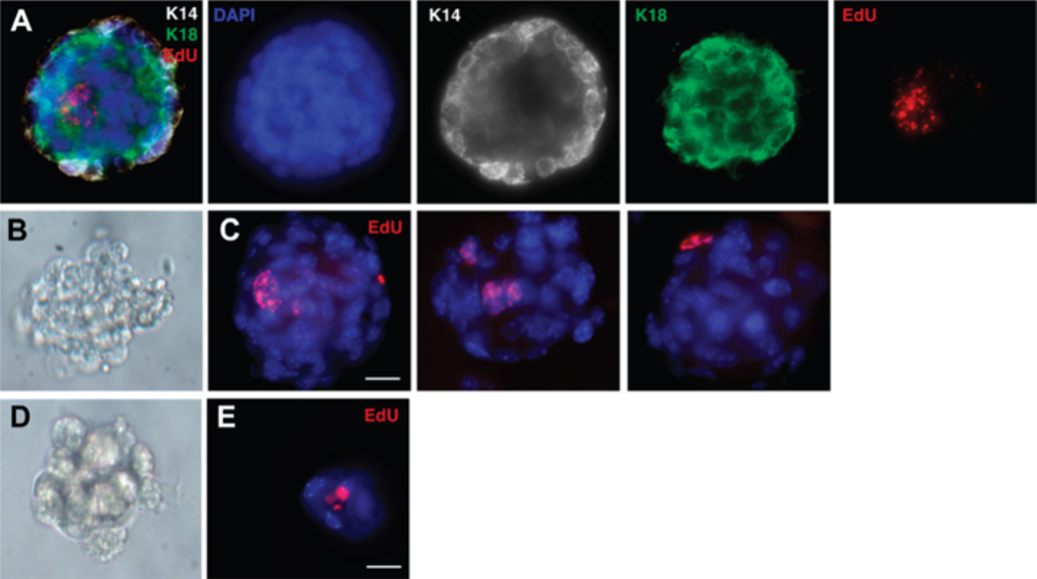
**eLLRCs from the MaSC population generate multi-lineage mammospheres. (A)** Three-dimensional image of mammosphere stained for EdU (red), K14 (white) and K18 (green). Representative bright field images of gross morphology of primary **(B)** and secondary **(D)** mammospheres grown under ultra low non-adherent conditions *in vitro*. **(C** **,** **E)** Immunofluorescence analysis of paraffin sections of mammospheres stained for EdU (red) with nuclear counterstain, DAPI (blue). C represents three different primary mammospheres while E is a second-generation mammosphere. DAPI, 4′,6-diamidino-2-phenylindole; EdU, 5-ethynl-2′-deoxyuridine; eLLRCs, embryonically derived long label retaining cells; MaSC, mammary stem cell.

Our data are consistent with the possibility that eLLRCs act as long-term stem/progenitor cells in the adult mammary gland. Two critical features of mammary stem/progenitor cells are that they give rise to all the cells within the gland and self-renew. The traditional assessment of these properties is the ability to repopulate the mammary gland in serial transplantation experiments. Unfortunately, the detection method for EdU requires fixation and permeabilization of cells for label visualization precluding their use in subsequent transplantation studies. Instead, to facilitate functional studies of eLLRCs, we performed *in vitro* MaSC assays [27],[28]. An alternative test of mammary ‘stemness’ is the ability to give rise to mammospheres containing both epithelial lineages when cells are cultured under non-adherent conditions. The assay is based on the premise that, under these culture conditions, the vast majority of differentiated cells dye by anoikis, but that stem/progenitor cells can survive and give rise to mammospheres, which are, themselves, enriched in mammary stem/prognitor cells. Given that eLLRCs were enriched in the basal population, we harvested MECs from eight-week-old mice that had been labeled with EdU during embryogenesis and isolated the CD24^+^CD49f^high^ basal cell fraction using FACS as previously described. However, we could not label for EdU without killing the cells, so we plated the sorted MaSC population at low density in 1% methylcellulose to inhibit cell aggregation. These conditions have previously been shown to ensure that each mammosphere is derived from a single cell [28]. Once formed, primary mammospheres were fixed, paraffin-embedded and analyzed for EdU labeling in serial sections, or they were dissociated and passaged in culture to form secondary mammospheres, which were also analyzed for EdU labeling after seven days. As shown in Figure 6, EdU-labeled cells were present in both primary and secondary mammospheres. Given the label dilution and rarity of cells in secondary mammospheres, further propagation of the LLRCs in third generation mammospheres was not evaluated. Importantly, in mammospheres with EdU^+^ cells, both K14 and K18 positive cells were detected. These results are consistent with the possibility that an individual eLLRC can give rise to an EdU-positive mammosphere and daughter cells of both luminal and basal lineages. However, since we could not sort for individual EdU-positive cells before plating, our results are not definitive. Nonetheless, the presence of EdU-positive cells within second-generation mammospheres suggests that the original eLLRCs were able to self renew during the formation of the primary mammospheres and give rise to subsequent mammospheres upon disaggregation and replating.

## Discussion

Cells in the embryonic bud give rise to all mammary epithelial cell lineages in the adult mammary gland. Furthermore, transplantation studies have demonstrated that a single embryonic mammary bud, harvested as early as e12, can repopulate an entire gland. Therefore, while it is obvious that these embryonic structures contain the precursors to adult MaSCs, it is not clear whether embryonic cells, themselves, can become long-lived, adult mammary stem/progenitor cells. In this study, we used DNA long label retention to ask whether cells from the embryonic mammary gland might contribute to the adult MaSC pool. We identified LLRCs originating during embryogenesis (eLLRCs) that localized to the nipple region in the primary ducts. Although enriched in the basal compartment, eLLRCs were found in both the myoepithelial and luminal epithelial cell layers. Our data support the functional capacity of eLLRCs as MaSCs *in vivo* and *in vitro*. We demonstrated that eLLRCs re-enter the cell cycle during puberty, pregnancy and tissue homeostasis, as well as in response to the exogenous administration of ovarian hormones. Furthermore, when cultured *in vitro*, eLLRCs contributed to the formation of primary and secondary mammospheres containing cells with both luminal and basal lineages, consistent with the idea that these cells were multipotent and able to self-renew. Therefore, eLLRCs demonstrate many characteristics of bi-potent stem/progenitor cells, suggesting that embryonic cells may directly contribute to a population of quiescent stem cells in the post-natal mammary gland.

We first examined whether cells in the embryonic mammary bud were bi-potential in terms of their lineage marker expression. We found that all cells within mammary placodes and mammary buds expressed the myoepithelial markers, K14 and p63, as well as the luminal markers, Gata3 and K18. The first indication of lineage separation was the restriction of p63 expression to the basal compartment at the initiation of ductal outgrowth. However, during the developmental stages that we examined, the expression of the other markers was heterogeneous and many cells expressed both K14 and either Gata3 and/or K18. Lineage markers were better segregated into their ultimate basal and luminal patterns at the distal ends of the growing ducts after birth suggesting that the establishment of the specific epithelial cell lineages may occur as a function of active proliferation and morphogenesis at the growth front, similar to what happens in TEBs during puberty. Other studies have reported that embryonic mammary epithelial cells express both luminal and basal lineages [12],[17],[31], but they have not defined the exact timing of lineage segregation. Our data suggest that the interval between the initiation of ductal outgrowth and birth is a dynamic period during which lineage specification and the acquisition of stem/progenitor activity occurs concurrent with the initiation of ductal morphogenesis.

Nuclear label retention for extended periods in individual cells may be due to the presence of labeled cells that differentiated and exited the cell-cycle shortly after the label was administered or may represent quiescent cells that divide infrequently or traverse the cell cycle very slowly. We labeled cells at the onset of proliferation and ductal morphogenesis in female embryos, from e14.5 to e18, a period during which Spike *et al.* recently demonstrated an increase in functional stem cell activity defined by transplantation studies [17]. We reasoned that cells from the mammary bud divide and give rise to a pool of mammary stem cells some of which become quiescent at birth and contribute to the development and homeostasis of the mammary gland over its lifespan. Our data support this model. We found eLLRCs that persisted for long periods of time in the mammary ducts and that divided in response to a variety of developmental and hormonal cues, ruling out that label-retaining cells were merely long-lived terminally differentiated cells. Furthermore, it appears that these cells have stem/progenitor capacity since they contributed to the formation of primary and secondary mammospheres, which contain both basal and luminal lineages. These data are consistent with previous cell sorting and lineage tracing studies demonstrating that embryonically derived cells can contribute to stem/progenitor cell compartments during ductal development [12],[13],[17]. Furthermore, our data also suggest that some eLLRCs divide and contribute to alveolar morphogenesis during pregnancy. These cells may be akin to basal stem/progenitor cells that contribute to alveolar development identified in lineage tracing experiments [13],[14].

Our analysis revealed that eLLRCs are limited to the primary ducts near the nipple region of the mammary gland. These cells were present in both basal and luminal populations as demonstrated by immunofluorescence and FACs analysis. Previous lineage tracing studies have suggested that embryonic K14-positive cells as well as Axin2-positive cells from the early embryonic mammary bud can give rise to luminal and/or myoepithelial cells during pre-pubertal ductal development [12],[13]. However, while these authors suggested that bipotent stem cells marked in the embryo do not persist in the postnatal animal [12], our studies provide evidence for a population of embryonically marked long-lived cells that are reactivated during subsequent stages of mammary development. It is likely that we labeled cells during the initial ductal outgrowth that contribute to multi-potent basal stem cells [see Additional file 2: Figure S2] as well as to unipotent basal and/or luminal progenitor pools created just prior to birth. Future studies combining lineage tracing techniques with embryonic DNA labeling will be necessary to determine whether eLLRCs represent the early unipotent stem cells created by birth [12], the bipotent MaSC confirmed to exist in the adult mammary gland [17] or, perhaps, both.

The location of eLLRCs near the nipple region is very similar to the location of LGR5 positive (*Lgr5* ^*+*^) cells within the gland. *Lgr5* ^+^ cells are a subset of K14-positive cells in the pubertal mammary gland [36],[37] that can efficiently regenerate a complete mammary epithelium upon transplantation of a single cell [36],[37]. These transplantation studies are further supported by recent lineage tracing that demonstrated Lgr5 labels multipotent stems cells as these cells give rise to both basal and luminal cell lineages [14]. The relationship of basal eLLRCs to *Lgr5* ^*+*^ cells will need to be explored in future studies. In particular, it is not clear why *Lgr5* ^*+*^ cells are limited to the nipple region, but we hypothesize that eLLRCs are found only within this region of the gland due to rapid dilution of the label as stem/progenitor cells proliferate symmetrically at the growth front. Those cells that retain label for long periods are likely those stem/progenitor cells that become quiescent behind the growth front. Alternatively, as suggested by Cairns and Smith [21],[38], stem/progenitor cells may divide asymmetrically, preserving a labeled ‘template’ DNA strand through multiple cell divisions, resulting in the retention of EdU for prolonged periods of time. However, previous transplantation studies have demonstrated that stem cells are present throughout the mammary ducts. If preservation of the template strand were present at the growth front, then one might have expected to see a wider distribution of EdU-labeled cells in our studies. Therefore, we believe that our results are more consistent with the dilution of label in TEBs due to symmetrical division of the cells. Of course, it is entirely possible that asymmetric division with preservation of the labeled template DNA strand contributes to gland homeostasis by the quiescent stem cells deposited behind the active growth front.

Previous studies have described LLRCs in the adult mammary gland. These experiments utilized tritiated thymidine and BrdU incorporation as well as transgenic H2B labeling in the adult mammary gland, during puberty and in the setting of transplantation. Our results with BrdU incorporation generally agree with previous findings in identifying a small percentage of label-retaining cells that are located in both the luminal and basal compartments and that express ERs and PRs [18]-[22],[24],[39]. Similar to our double labeling experiments, Smith found that LLRCs in transplanted glands could re-enter the cell cycle upon hormonal stimulation [21]. Our results suggest that LLRCs labeled during puberty are generally located in the middle of the gland and we did not find these cells at the leading edges of the ducts. Previous studies also labeled during puberty but only one mentioned a specific distribution pattern of label-retaining cells [20],[22],[39]. Multi-scale *in-situ* analysis revealed that the ventral-most, large ducts near the nipple region contained a reservoir of undifferentiated, putative stem cells similar to our studies, although the LLRCs were not restricted to this region in their study [22]. The two-week labeling protocol in each of these studies encompassed a much longer period of ductal elongation than in our experiments and may have widened the distribution of LLRCs. In addition to confirming these previous studies [20],[22],[39], we have identified an embryonically derived stem/progenitor cell population that is activated both during puberty and pregnancy. A small number of the eLLRCs that also incorporate BrdU persist as long-term double-labeled cells. However, whether these double-labeled cells represent the same population as the previously reported adult LLRCs remains unknown [20],[22],[39]. Future gene expression profiling of double-labeled EdU^+^BrdU^+^ eLLRCs may shed light on this question and could enable the identification of more precise markers for quiescent stem cells.

## Conclusions

In conclusion, we find a population of embryonically labeled cells that become quiescent and are called upon during active phases of hormonally regulated postnatal mammary gland development and remodeling. These cells are bipotent and can contribute to both luminal and myoepithelial cells in culture. The location of these putative embryonically derived stem/progenitor cells within the ductal tree as well as our serial double labeling experiments suggest that quiescent stem cells are likely deposited along the ductal tree just behind the active growth front. Breast stem cells contribute to all major stages of breast development, actively maintain breast tissue for the life of the individual and may be important targets for transformation. Therefore, further characterization of how embryonically-derived cells contribute to the stem/progenitor pool will help to unlock basic mechanisms by which stem cells are generated and maintained. Furthermore, embryonic exposures have been shown to increase the risk of breast cancer [40],[41]. Therefore, the presence of embryonically-derived stem cells in the adult gland may help to explain how these fetal risks manifest in breast cancer much later in life and it will be important to understand how embryonic LLRCs respond to embryonic insults that have been shown to increase cancer risk.

## Additional files

## Electronic supplementary material

Additional file 1: Figure S1.: EdU long label retention is found within cells of the basal and luminal lineages. Immunofluorescence analysis of lineage markers in mammary glands from eight-week-old mice that had been administered EdU between e14.5 and e18.5. Paraffin sections were co-stained for EdU and the myoepithieial markers K14 **(A)**, and p63 **(B)**, or the luminal markers, Gata3 **(C)**, estrogen receptor, ER **(D)** and progesterone receptor, PR **(E)**. Boxes denote the portion of the ducts shown in the magnified images. L denotes the luminal aspect of the duct surface. Scale bars represent 20 μm. (JPEG 5 MB)

Additional file 2: Figure S2.: EdU-positive cells can be detected after labeling of embryonic mammary epithelial cells. **(A)** Representative FACS dot plots of live lineage depleted, basal and luminal epithelial cells used for isolation to detect EdU label retention. Representative histograms showing EdU labeling in FACS isolated epithelial cells from mammary glands of mice injected with EdU 2X daily at **(B)** e14 alone, **(C)** e15 alone, **(D)** e16 alone and **(E)** from e14 to e18. (JPEG 1 MB)

Below are the links to the authors’ original submitted files for images.

Authors’ original file for figure 1

Authors’ original file for figure 2

Authors’ original file for figure 3

Authors’ original file for figure 4

Authors’ original file for figure 5

Authors’ original file for figure 6

Authors’ original file for figure 7

Authors’ original file for figure 8

## Abbreviations

%: percentage lumen
bFGF: basic fibroblast growth factor
BrdU: 5-bromo-2′-deoxyuridine
(D)MEM: (Dulbecco’s) modified Eagle’s medium. e, embryonic
E: estradiol
EDTA: ethylenediaminetetraacetic acid
EdU: 5-ethynl-2′-deoxyuridine
EGF: epidermal growth factor
ER: estrogen receptor
FACS: fluorescence activated cell sorting
FBS: fetal bovine serum
H2B-GFP: histone 2b-green fluorescent protein
K14: keratin 14
K18: keratin 18
K5: keratin5
Lgr5: leucine-rich repeat containing G protein-coupled receptor 5
Lin: lineage depleted
LLRC: long label retaining cell
MaSC: mammary stem cell
MECs: mammary epithelial cells
MG: mammary gland
mm: mammary mesenchyme
N: Number
P: progesterone
PFA: paraformaldehyde
PR: progesterone receptor
S: supplementary
SE: standard error
SEM: standard error of the mean
TEBs: terminal end buds
tTA: tetracycline transactivator
