# Supplementary figures and images for: Embryonic cells contribute directly to the quiescent stem cell population in the adult mouse mammary gland
Source: Breast Cancer Res. 2014 Dec 3;16:487. doi: 10.1186/s13058-014-0487-6 (PMC4308878; doi:10.1186/s13058-014-0487-6)

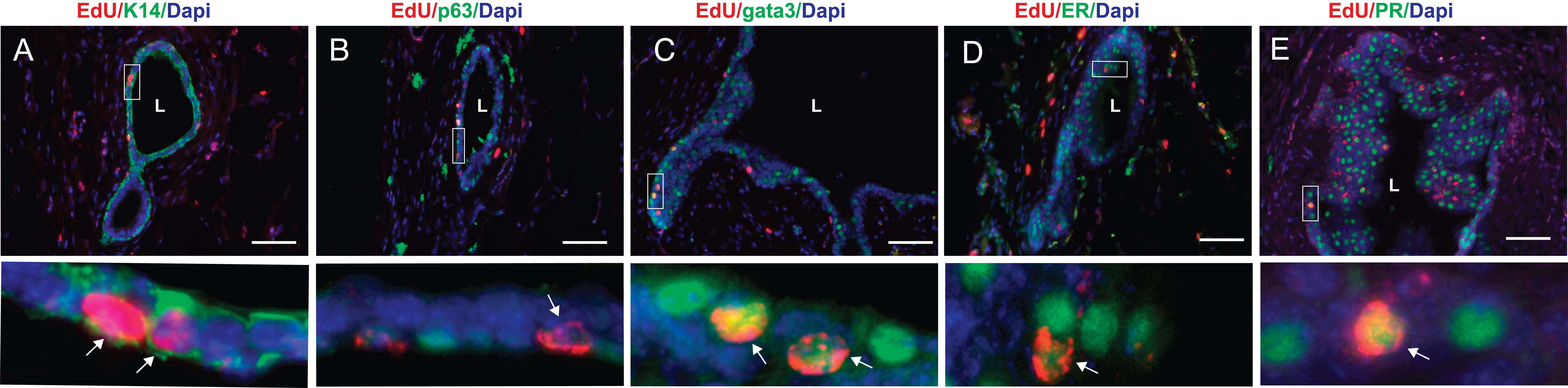

Supplement: Supplementary file 1 — Additional file 1: Figure S1.: EdU long label retention is found within cells of the basal and luminal lineages. Immunofluorescence analysis of lineage markers in mammary glands from eight-week-old mice that had been administered EdU between e14.5 and e18.5. Paraffin sections were co-stained for EdU and the myoepithieial markers K14 (A), and p63 (B), or the luminal markers, Gata3 (C), estrogen receptor, ER (D) and progesterone receptor, PR (E). Boxes denote the portion of the ducts shown in the magnified images. L denotes the luminal aspect of the duct surface. Scale bars represent 20 μm. (JPEG 5 MB) [file 13058_2014_487_MOESM1_ESM.jpeg]

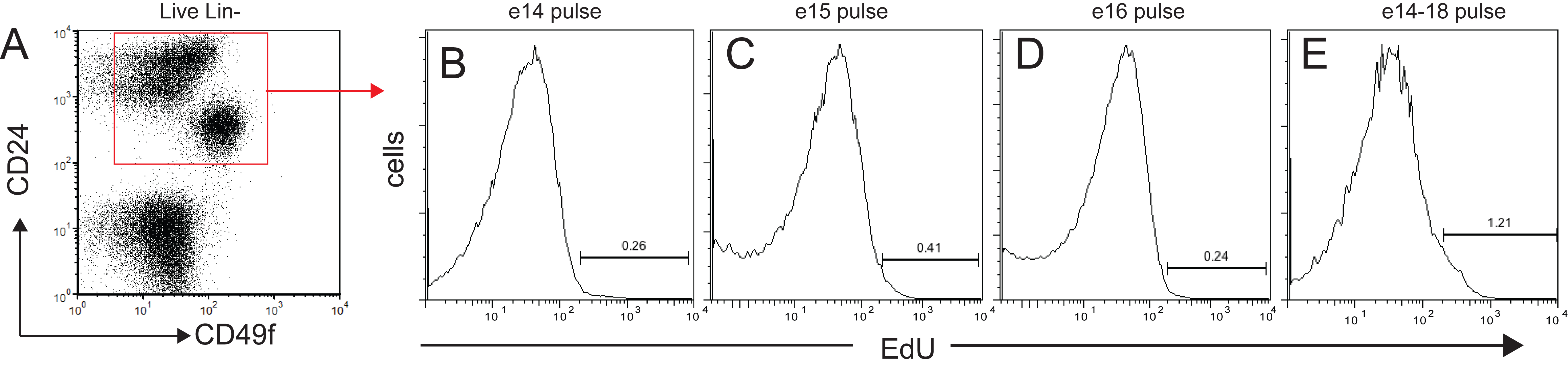

Supplement: Supplementary file 2 — Additional file 2: Figure S2.: EdU-positive cells can be detected after labeling of embryonic mammary epithelial cells. (A) Representative FACS dot plots of live lineage depleted, basal and luminal epithelial cells used for isolation to detect EdU label retention. Representative histograms showing EdU labeling in FACS isolated epithelial cells from mammary glands of mice injected with EdU 2X daily at (B) e14 alone, (C) e15 alone, (D) e16 alone and (E) from e14 to e18. (JPEG 1 MB) [file 13058_2014_487_MOESM2_ESM.jpeg]

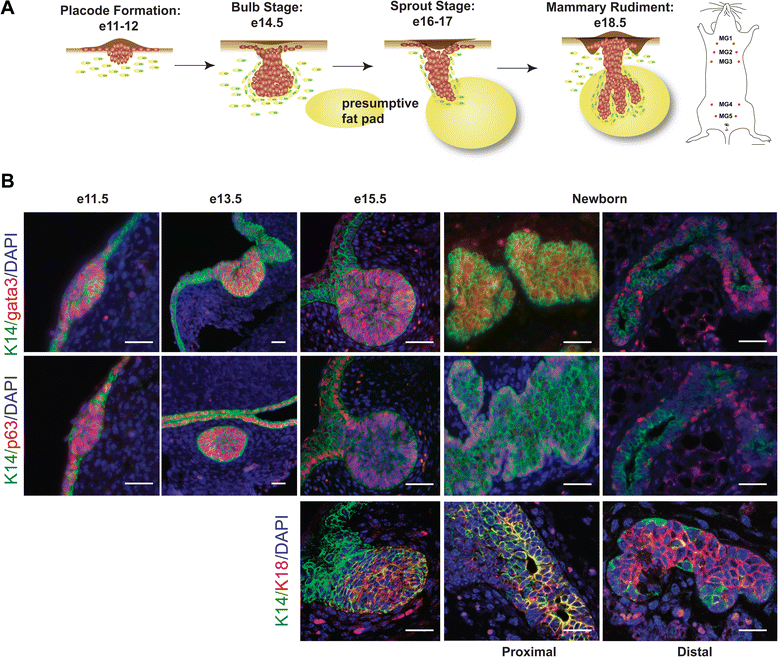

Supplement: Supplementary file 3 — Authors’ original file for figure 1 [file 13058_2014_487_MOESM3_ESM.gif]

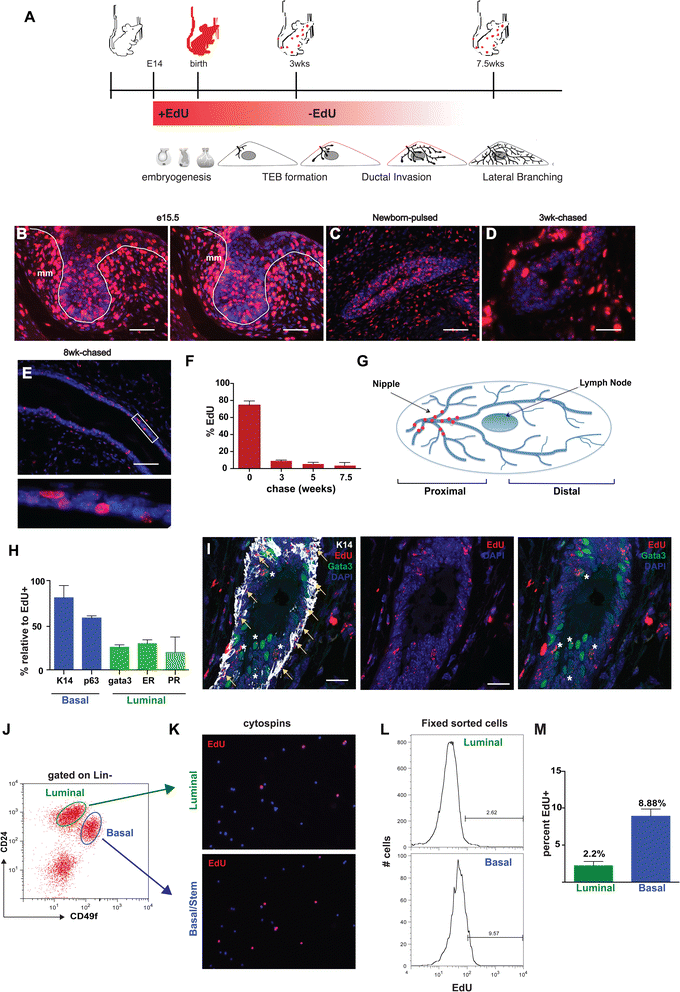

Supplement: Supplementary file 4 — Authors’ original file for figure 2 [file 13058_2014_487_MOESM4_ESM.gif]

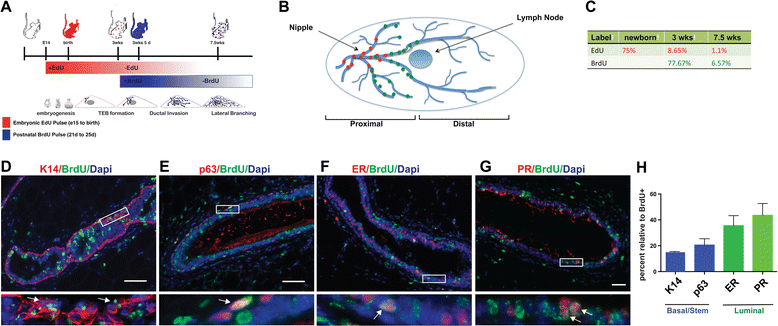

Supplement: Supplementary file 5 — Authors’ original file for figure 3 [file 13058_2014_487_MOESM5_ESM.gif]

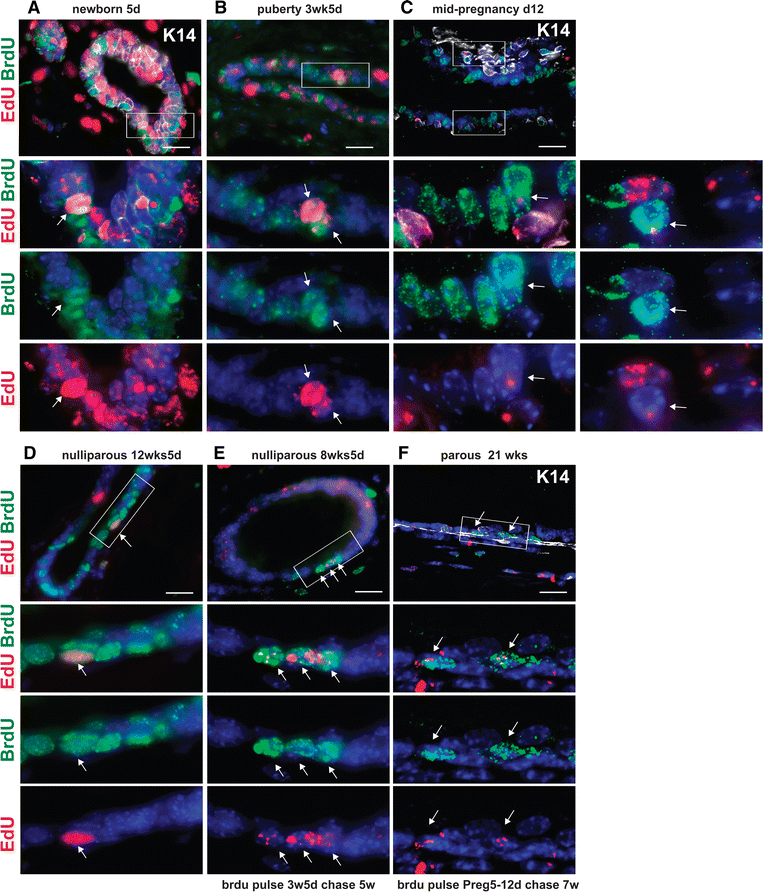

Supplement: Supplementary file 6 — Authors’ original file for figure 4 [file 13058_2014_487_MOESM6_ESM.gif]

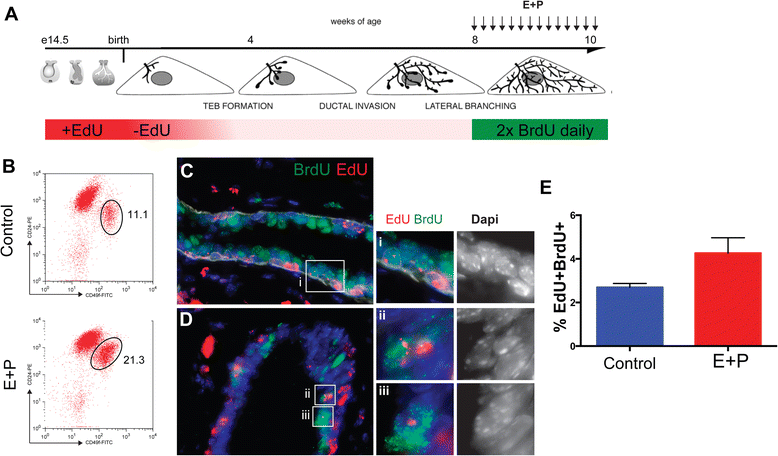

Supplement: Supplementary file 7 — Authors’ original file for figure 5 [file 13058_2014_487_MOESM7_ESM.gif]

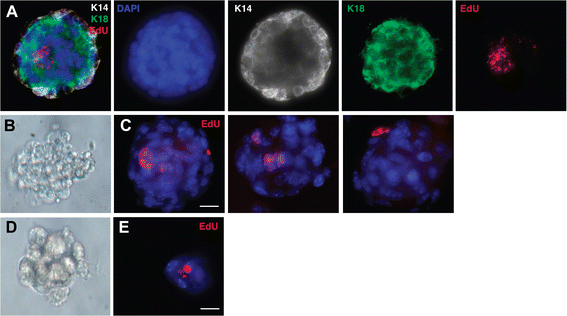

Supplement: Supplementary file 8 — Authors’ original file for figure 6 [file 13058_2014_487_MOESM8_ESM.gif]
